# The Development of Lipid-Based Sorafenib Granules to Enhance the Oral Absorption of Sorafenib

**DOI:** 10.3390/pharmaceutics15122691

**Published:** 2023-11-28

**Authors:** Jaylen C. Mans, Xiaowei Dong

**Affiliations:** Department of Pharmaceutical Sciences, University of North Texas Health Science Center, Fort Worth, TX 76107, USA

**Keywords:** oral formulation, lipid-based formulations, poorly water-soluble drug, bioavailability enhancement, anticancer

## Abstract

Sorafenib (SFN) is an anticancer multi-kinase inhibitor with great therapeutic potential. However, SFN has low aqueous solubility, which limits its oral absorption. Lipids and surfactants have the potential to improve the solubility of water-insoluble drugs. The aim of this study is thus to develop novel lipid-based SFN granules that can improve the oral absorption of SFN. SFN powder was coated with a stable binary lipid mixture and then absorbed on Aeroperl 300 to form dry SFN granules with 10% drug loading. SFN granules were stable at room temperature for at least three months. Compared to SFN powder, SFN granules significantly increased SFN release in simulated gastric fluid and simulated intestinal fluid with pancreatin. Pharmacokinetics and tissue distribution of SFN granules and SFN powder were measured following oral administration to Sprague Dawley rats. SFN granules significantly increased SFN absorption compared to SFN powder. Overall, the lipid-based SFN granules provide a promising approach to enhancing the oral absorption of SFN.

## 1. Introduction

Sorafenib (SFN) is a promising anticancer therapeutic agent approved by the Food and Drug Administration (FDA) for the treatment of patients with advanced hepatocellular carcinoma, advanced renal cell carcinoma, and differentiated thyroid cancer. SFN works as a multi-kinase tyrosine inhibitor, with activity disrupting RAF, vascular endothelial growth factor receptor (VEGFR), and platelet-derived growth factor receptor (PDGFR) kinases [1,2]. The broad pharmacodynamic activity against RAF, VEGFR, and PDGFR kinases provides SFN with extensive antiangiogenic and antitumor proliferative activity against a range of cancers [1,2]. However, SFN is highly lipophilic (LogP 3.8) and insoluble in water (<25 ng/mL). The commercially available sorafenib oral tablet, Nexavar, contains crystalline sorafenib tosylate, a salt form to improve solubility; however, sorafenib tosylate is still insoluble (only 60 μg/mL in water). As a biopharmaceutical classification system class II compound, SFN exhibits poor water solubility, which restricts its absorption into the systemic circulation [3,4]. In addition to poor water solubility, SFN undergoes extensive first-pass metabolism, further reducing its bioavailability [5,6,7]. Clinicians must use frequent high-dosage administration of SFN (400 mg per day) to overcome the low solubility and extensive first-pass metabolism of SFN. The usage of a high dose of SFN could contribute to toxicity observed in patients such as hand–foot skin reactions, hypertension, and gastrointestinal issues, as well as lesser reported issues [8,9,10,11]. These toxicities can be poorly tolerated by patients, often leading to clinical dose management or complete treatment discontinuation [12,13,14]. With poor oral bioavailability and low tissue exposure levels, SFN is restricted in terms of its therapeutic potential as an oral anti-cancer agent [3,15,16]. Consequently, researchers have been investigating alternative formulation strategies to overcome the poor water solubility of SFN, thereby increasing the oral bioavailability and improving the therapeutic outcome. Improved SFN formulations could produce the same therapeutic effect at lower doses, thereby reducing the associated toxicity risk and presumably increasing patient satisfaction.

Lipid-based formulations such as solid lipid nanoparticles, microemulsions, and liposomes have the potential to solve the solubility issues of poorly water-soluble drugs. They are prepared from biodegradable and biocompatible lipids and surfactants, reducing likelihood of excipient-safety concerns. However, nanoparticles commonly are made in aqueous phases. Nanoparticles naturally tend to aggregate together over time during storage to decrease their free energy, which can lead to erratic changes in particle behavior and stability [17]. Other lipid-based formulations such as microemulsions and emulsions are also prepared as liquids, leading to issues with stability, the manufacturing process, and costs. Conversion of liquid lipid-based formulations to solid dosage forms is desirable; however, low drug loading (DL) in final solid forms, complex drying procedures, and size increase after drying have hindered such conversion for oral medication formulations. To overcome these issues and use lipid-based formulations in solid dosage forms for oral administration, our lab discovered a new approach to preparing lipid-based solid drug granules with high DL [18,19,20,21]. Recently, we confirmed that the binary lipid mixture of Miglyol 812 and D-α-tocopheryl polyethylene glycol 1000 succinate (TPGS) we used in the granules formed stable particles that were not impacted by water dilution in a pseudo-ternary phase diagram [20].

In this study, we aimed to prepare novel lipid-based SFN granules to enhance the oral absorption of SFN. We investigated if coating SFN with the stable binary lipid mixture of Miglyol 812 and TPGS will increase oral absorption. The novel SFN granules were characterized by stability in simulated fluids, two-step biorelevant dissolution, physical state, long-term stability, and in vivo studies, including pharmacokinetics and tissue distribution.

## 2. Methods

### 2.1. Materials

Research-grade SFN free base was purchased from LC Laboratories (Woburn, MA, USA). TPGS was provided as a gift from BASF (Ludwigshafen, Germany). Miglyol 812 (middle-chain triglycerides) was obtained as gifts from Cremer (Eatontown, NJ, USA). Aeroperl 300 (colloidal silicon dioxide) was provided as a gift from Evonik (Parsippany, NJ, USA). Amicon Ultra-0.5 centrifugal filter unit with a molecular weight cutoff of 100 kDa was purchased from Millipore (Bedford, MA, USA). HPLC-grade methanol was purchased from Fisher Scientific (Fair Lawn, NJ, USA).

### 2.2. Animals

Sprague Dawley rats (males, 276–300 g) were purchased from Charles River Laboratories (Wilmington, MA, USA). All animal experiments were carried out under an approved protocol by the Institutional Animal Care and Use Committee at the University of North Texas Health Science Center on 27 March 2020. Rats were housed in groups of 2 under a 12 h light/dark cycle with free access to food and water for one week before use.

### 2.3. Preparation of SFN Granules

SFN granules were prepared at 10% DL. Briefly, 16.8 mg of SFN free base, 50 mg of Miglyol 812, and 50 mg of TPGS were weighed into a glass vial. The mixture was stirred at 50 °C for 15 min. Next, 50 mg of Aeroperl 300 was incrementally added to the vial. After the mixture was thoroughly homogenized, the mixture was cooled to room temperature to form SFN granules.

### 2.4. Characterization of SFN Granules

#### 2.4.1. Differential Scanning Calorimetry and Fourier Transform Infrared Spectroscopy Analysis of SFN Granules

The physical state of SFN within SFN granules was evaluated by using differential scanning calorimetry (DSC) and Fourier transform infrared spectroscopy (FTIR) analysis. DSC analysis of SFN granules was performed using a PerkinElmer DSC 4000. Briefly, samples were sealed inside an aluminum DSC pan and equilibrated to 20 °C for 1 min. Next, samples were heated along a heat curve of 10 °C/min from 20 °C to 240 °C. Relative heat flow was recorded for comparative analysis. SFN granules were also analyzed via Thermo Scientific Nicolet iS5 FTIR spectrometer to measure infrared transmittance from 500 cm^−1^ to 3700 cm^−1^. The fingerprint FTIR spectra of SFN granules, blank granules, unprocessed physical mixtures, and SFN free base were compared for analysis. Blank granules were prepared by following the procedure in Section 2.3 without SFN.

#### 2.4.2. Measurement of Drug Loading in SFN Granules

DL in SFN granules was measured by HPLC. Briefly, SFN granules were dissolved in methanol and then centrifuged for 5 min at 15,000 rpm. Following centrifugation, 200 μL of supernatant was collected for HPLC analysis as previously reported [22]. The experiments were conducted in triplicate. DL was calculated as follows:

% DL = [(drug in the granules)/(total weight of drug granules)] × 100% (*w*/*w*)

### 2.5. Characterization of Particles Released from SFN Granules

#### 2.5.1. Determination of Particle Size and Size Distribution

SFN granules were suspended in Milliq water. The mixture was vortexed for 30 s and centrifuged for 5 min at 15,000 rpm at room temperature. The supernatant was measured for particle size and size distribution by a dynamic light scattering system (Malvern Zetasizer Ultra Particle Analyzer).

#### 2.5.2. Determination of the Percentage of SFN Entrapped in Particles

After SFN-loaded particles (the supernatant as described above) were collected, the total drug content in the particles was measured by HPLC. To measure how much SFN was entrapped in the particles, free SFN in the SFN-loaded particles was separated using an Amicon Ultra-0.5 centrifugal filter unit with a molecular weight cutoff of 100 KD. The filter was pretreated with a solution containing 0.2% Tween 80 and 0.9% NaCl to prevent the binding of SFN to the filter membrane. The experiments were conducted in triplicate. The concentration of SFN in the filtrate was measured by HPLC. The percentage of SFN entrapped in the particles was calculated as follows:

% entrapped SFN = [1 − (free SFN/total SFN in the particles)] × 100% (*w*/*w*)

#### 2.5.3. Evaluation of Morphology for SFN-Loaded Particles

SFN-loaded particles were imaged using an FEI Tecnai G^2^ Spirit Transmission Electron Microscope (TEM) equipped with a LaB6 source at 120 kV using a Gatan ultrascan CCD camera. The SFN samples were prepared for imaging with the following procedure: Formvar carbon grids were placed on a glass slide and glow-discharged. Next, SFN-loaded particles were diluted with water (10:90, *v*/*v*), and 2.5 μL of diluted SFN-loaded particles was placed onto the grid and dried for 30 min. Then, 2.5 μL of uranyl acetate was applied to the grid and left to dry for 30 min. The mounted grids were loaded into TEM for imaging.

### 2.6. Short-Term Particle Size Stability of SFN-Loaded Particles at 37 °C in Physiologically Relevant Media

The two-step media including simulated gastric fluid (SGF, pH 1.2) and simulated intestinal fluid (SIF, pH 6.8) were prepared as previously reported [23]. A total of 12 mg of SFN granules were added into 11 mL of SGF, pH 1.2 at 37 °C, stirring at 150 rpm. At 0, 1, and 2 h, 1.5 mL media were withdrawn and centrifuged at 15,000 rpm for 10 min at room temperature. Following centrifugation, 1 mL of supernatant was collected into a cuvette for particle size analysis as described in Section 2.5.1. Immediately following the 2 h collection interval, the SGF medium was replenished to its initial volume and adjusted to pH 6.8 by adding 200 μL of 2 M KH_2_PO_4_ and 2.5 mL of 0.5 M NaOH to mimic SIF. Subsequently, at 4, 6, and 8 h, samples were withdrawn for particle size measurement as described above. The experiments were conducted in triplicate.

### 2.7. Long-Term Stability of SFN Granules

The long-term stability of SFN granules was assessed at room temperature. Parameters of particle size, DL, and entrapped SFN in the particles were measured as described above over three months. The degradation of SFN was monitored according to the appearance of extra peaks on HPLC chromatograms. Three independent batches of SFN granules were prepared and monitored for long-term stability.

### 2.8. In Vitro Dissolution Studies

The two-step dissolution of SFN granules and SFN powder was conducted as previously reported [23]. Briefly, 50 mg of SFN granules was added to 24 mL of SGF medium and stirred at 225 rpm at 37 °C. At 0, 15 min, 30 min, 1 h, 1.5 h, and 2 h, 1 mL of sample was withdrawn and centrifuged at 15,000 rm for 5 min at room temperature. After centrifugation, 200 μL of supernatant was diluted with methanol, vortexed for 1 min, and then centrifuged at 15,000 rpm for 5 min. After centrifugation, 200 μL of supernatant was collected for HPLC analysis as previously described. After withdrawing samples from SGF at 2 h, 0.3 mL 2M KH_2_PO_4_ and 16 mL of water were added to switch the media to SIF, and 2M NaOH was used to adjust pH to 6.8, and then 4 mL of pancreatin solution was added to the media. Sample collection was continued at 10 min, 20 min, 30 min, 45 min, 1 h, 2 h, 3 h, and 4 h. Drug concentrations were measured by HPLC as described above. SFN powder at an equivalent amount of SFN was tested as a control. The experiments were conducted in triplicate.

### 2.9. Pharmacokinetic Study

Sprague Dawley male rats (276–300 g, *n* = 3 per group) were randomly grouped and given SFN powder or SFN granules by oral gavage at 30 mg/kg of SFN. SFN powder and SFN granules were pre-mixed with water before dosing. After dose administration, blood samples were collected at 0, 0.5, 1, 2, 3, 4, 5, 6, 7, 8, 32, 48, and 72 h in EDTA-coated tubes. Blood samples were immediately centrifuged at 4000 rpm for 5 min at 4 °C to obtain plasma samples. Plasma samples were stored at −80 °C until further analysis within three months. SFN concentrations in plasma samples were measured by a previously reported LC-MS method [22].

### 2.10. Tissue Distribution Study

The tissue distribution of SFN granules and SFN powder was measured in Sprague Dawley male rats (276–300 g, *n* = 3 per group). Briefly, SFN powder and SFN granules, which were suspended in water, were orally administered to randomly divided rats at 30 mg/kg of SFN, respectively. After 2 h, rats were sacrificed to collect tissues including lung, mesenteric lymph node, liver, kidney, brain, heart, and spleen. Tissue samples were stored at −80 °C until further analysis within three months. SFN concentrations in tissue samples were measured by a previously reported LC-MS method [22].

### 2.11. Statistical Analysis

The results were expressed as mean ± standard deviation (SD). The data were compared using a Student *t*-test at a 95% confidence level, and *p* values < 0.05 were considered significantly different.

## 3. Results

### 3.1. Characterization of SFN Granules and SFN-Loaded Particles

**Figure 1:**
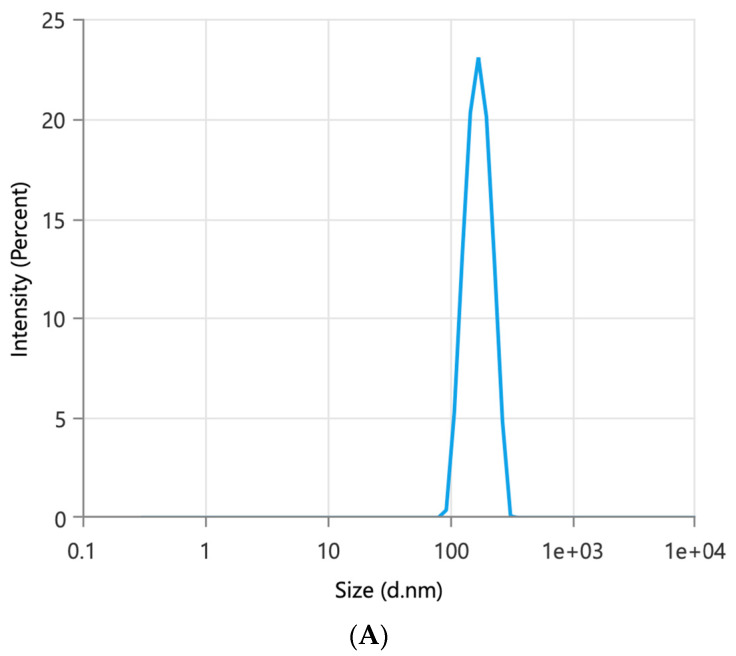

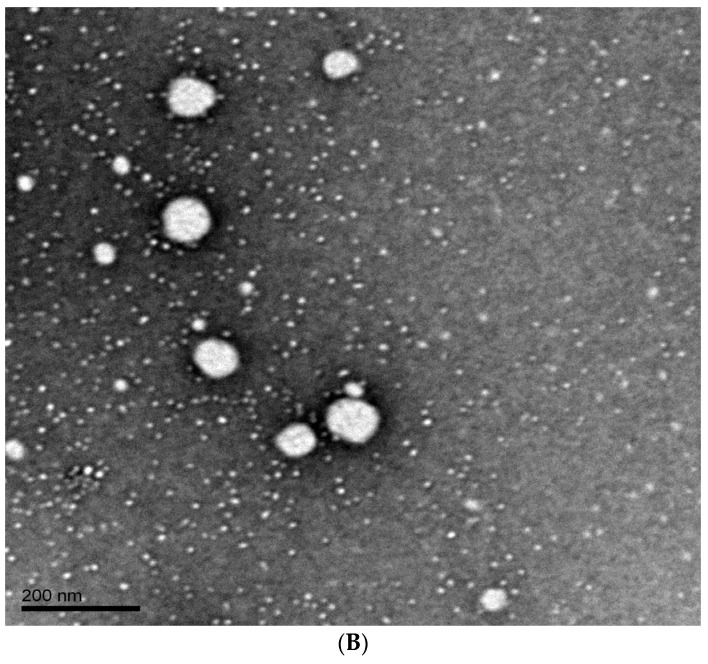
SFN granules produced SFN-loaded particles when introduced to contact with water. (**A**) Particle size and size distribution of SFN-loaded particles. (**B**) TEM image of SFN-loaded particles.

SFN granules were successfully prepared with a binary lipid mixture of Miglyol 812 and TPGS. As demonstrated by the previous study, Miglyol 812 and TPGS at a 1:1 ratio (*w*/*w*) formed a stable binary system that is resistant to physical changes caused by water dilution [20]. Aeroperl 300 was used as a solid carrier to prepare dry granules with a good flow. When the amount of Aeroperl 300 reached 30% of the total amount of the granule, the flow of the granules was good. SFN granules had 10% DL and showed good flow properties. Upon contact with water, SFN granules spontaneously produced SFN-loaded particles. The particle size of SFN-loaded particles was 154 nm with a monodispersed size distribution indicated by a polydispersity index (P.I.) of 0.27 (Figure 1A). A TEM image further demonstrated the formation and size of SFN-loaded particles as well as their spherical morphology (Figure 1B). As shown in Table 1, about 99% of SFN was entrapped in the particles. SFN granules remained stable over a 3-month measurement period in terms of DL% and degradation %. During the storage period, SFN granules produced stable SFN-loaded particles. Although the particle size of SFN-loaded particles increased in the second and third month, they were still below 200 nm.

### 3.2. Physical state of SFN in SFN Granules

**Figure 2:**
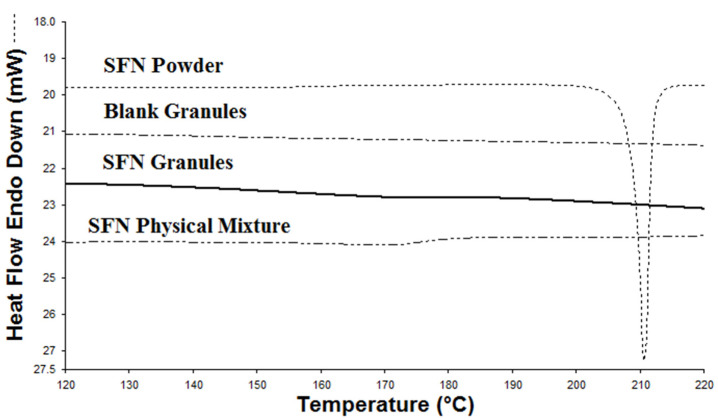
DSC differential thermograms of SFN powder, SFN granules, SFN physical mixture, and blank granules.

**Figure 3:**
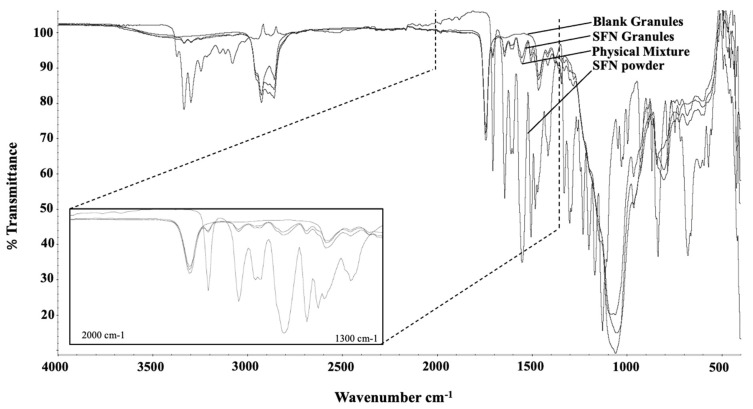
FTIR spectra of SFN powder, physical mixture, SFN granules, and blank granules. SFN was partially converted to an amorphous form.

The physical state of SFN in SFN granules was evaluated by DSC and FTIR analysis. In the DSC analysis, SFN powder displays a heat flow peak at 211.6 °C that was correlated to the melting point of SFN crystals. The melting point of SFN did not appear in blank granules nor SFN granules (Figure 2). To further test the physical state of SFN in the granules, FTIR analysis served as an alternative measurement for crystallinity to validate the DSC analysis. In FTIR analysis, both SFN powder and the physical mixture showed sharp characteristic peaks at 3300–3340 cm^−1^, 1550–1650 cm^−1^, and 670–680 cm^−1^, which were present, yet significantly reduced in SFN granules, and completely absent in the blank granules (Figure 3). Thus, according to the FTIR results, SFN was partially converted to an amorphous form in SFN granules. It is very likely, during the heating process in DSC measurement, that crystal SFN dissolved in the excipients, which resulted in the disappearance of the thermal peak in the physical mixture and SFN granules.

### 3.3. Short-Term Particle Stability of SFN-Loaded Particles in Physiological Conditions

**Figure 4:**
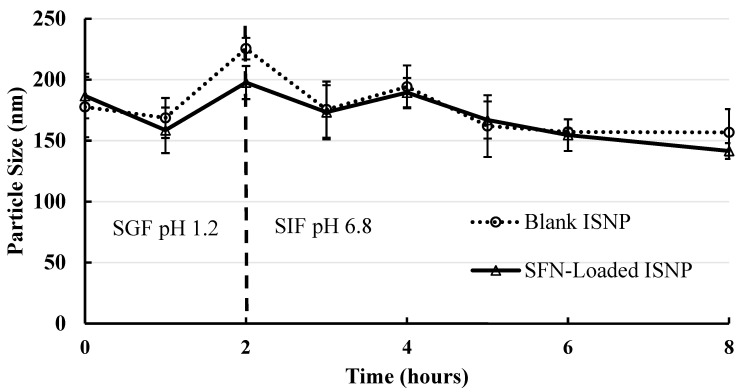
Short-term physical stability of SFN-loaded particles and blank particles in SGF for 2 h and SIF for following 6 h (*n* = 3). SFN-loaded particles were stable in SGF for 2 h and SIF for 6 h. Data are presented as mean ± SD.

The stability of SFN-loaded particles produced from SFN granules was assessed in SGF for 2 h to mimic the transition time in the stomach, followed by SIF for 6 h to mimic the transition time in the small intestine. As shown in Figure 4, the SFN-loaded particles maintained a narrow average size range of 143-198 nm, while the blank particles maintained a similar 157-226 nm average particle size range. There were no significant changes in the tested time points, compared to the size at time 0 for each group (Figure 4), indicating that SFN-loaded particles were stable in the tested physiological conditions.

### 3.4. Two-Step In Vitro Dissolution

**Figure 5:**
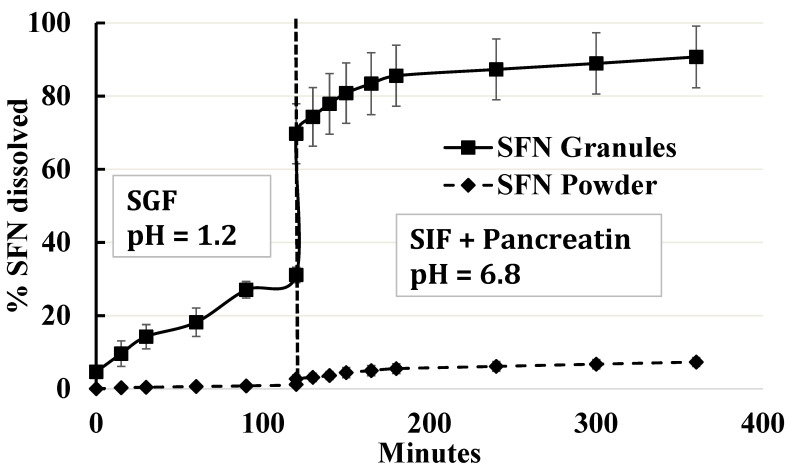
In vitro dissolution profile of SFN granules and SFN powder in SGF for 2 h, followed by SIF with the addition of pancreatin for 4 h (*n* = 3). SFN granules increased the dissolution of SFN compared to SFN powder. In SIF, about 90% of SFN was released from SFN granules. Data are presented as mean ± SD.

Dissolution studies were conducted over 2 h in SGF solution and 4 h in SIF in the presence of pancreatin. The current clinical single dose of SFN is 400 mg. To mimic SFN concentration in the stomach, 50 mg of SFN granules was used in the dissolution study in 24 mL media. As shown in Figure 5, about 27% of SFN was released from SFN granules, whereas about ~1% of SFN was released from SFN powder in the first 90 min. After the media were adjusted to SIF with pancreatin, the dissolution of SFN granules increased to 91% while SFN powder reached 7.3% (*n* = 3). Comparing SFN granules with SFN powder, there was a significant difference in the dissolution profiles in both the SGF and SIF with pancreatin media (*p* < 0.05).

### 3.5. Pharmacokinetics and Tissue Distribution

**Figure 6:**
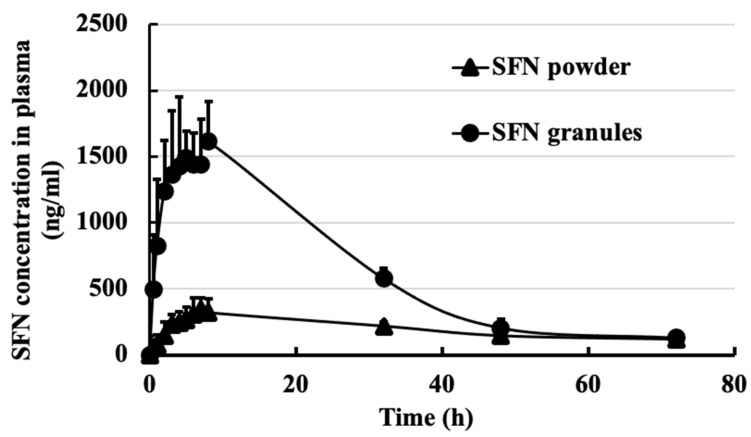
SFN plasma concentration in rats over 72 h following oral administration of SFN powder and SFN granules at 30 mg/kg (*n* = 3). SFN granules increased the SFN concentration in blood compared to SFN powder. Data are presented as mean ± SD.

**Figure 7:**
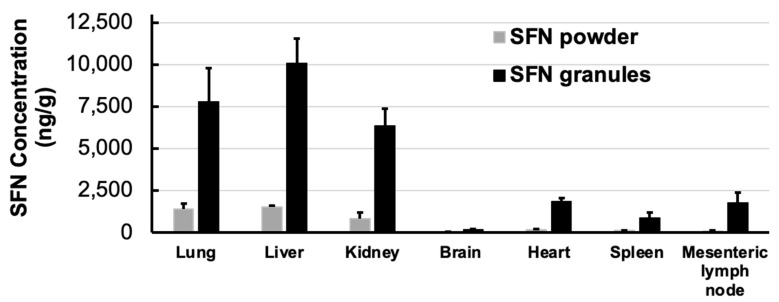
SFN concentrations in rat tissues following oral administration of SFN powder and SFN granules at 30 mg/kg at 2 h (*n* = 3). SFN granules increased SFN absorption over 6–10 fold except in brain. Particularly, SFN granules greatly increased drug concentration in mesenteric lymph node (over 20-fold) compared to SFN powder, suggesting the enhanced lymphatic uptake by SFN granules. Data are presented as mean ± SD.

The pharmacokinetic and biodistribution experiments were performed on Sprague Dawley rats. Pharmacokinetics measure drug concentrations in blood circulation, and biodistribution measures drug concentrations in each tissue. In the pharmacokinetic study, SFN granules demonstrated an over 4-fold increase in C_max_ compared to SFN powder (*p* < 0.05) (Figure 6). For tissue distribution, SFN granules significantly increased SFN uptake in all measured tissues by 6–10 fold, excluding brain tissue (Figure 7). Importantly, in mesenteric lymph nodes, SFN granules demonstrated a 20-fold increase in SFN concentration compared to SFN powder (Figure 7).

## 4. Discussion

SFN was the first molecular-targeted agent to be licensed for metastatic renal cell carcinoma in 2005 and later for unresectable hepatocellular carcinoma and advanced thyroid carcinoma. SFN is hydrophobic and has poor water solubility. Lipid-based formulations present an emerging mechanism to enhance drug absorption. However, conventional lipid-based formulations are not in solid forms and are filled in soft gelatin capsules [19]. To prolong shelf-life and improve the manufacturing process, the conversion of lipid-based formulation to solid forms has been studied. However, low DL and complicated processes were problematic. Here, we prepared SFN granules by coating SFN with a stable binary mixture composed of Miglyol 812 and TPGS. The DL of SFN granules was 10%. To understand the behavior of SFN granules upon contact with water, the particles in the supernatant after SFN granules were dispersed into the water were tested. The particle size and TEM data showed that after mixing with water, SFN granules generated SFN-loaded particles with high entrapment (Table 1 and Figure 1). In the granule preparation, the surface of SFN powder was coated with Miglyol 812 and TPGS, and then the coated SFN was absorbed on Aeroperl 300 to form dry SFN granules. Since SFN is water-insoluble, when SFN granules were mixed with water, Miglyol 812 and TPGS were released from Aeroperl 300 and formed stable particles by self-assembly, while SFN dissolved and was entrapped into Miglyol 812-TPGS particles. In this study, SFN granules were stable for three months in measured parameters without significant degradation. During the stability testing, SFN granules produced SFN-loaded particles without changes in particle size, size distribution, or the percentage of entrapped SFN (Table 1). The FTIR studies demonstrated that the crystal SFN was partially converted to an amorphous form in SFN granules, which could be caused by the interaction of the binary lipid mixture with the crystal SFN during the preparation.

One of the features of the binary lipid mixture of Miglyol 812 and TPGS is that they form stable particles in different physiological conditions. As demonstrated by the previous pseudo-ternary phase diagram, Miglyol 812 and TPGS formed a stable binary structure at a 1:1 ratio that allows it to maintain its structural integrity over water dilution [20]. Here the particle size of SFN-loaded particles was stable in SGF (pH 1.6) for 2 h and SIF (pH 6.8) for 4 h (Figure 4). The strong interaction among Miglyol 812, TPGS, and SFN could provide structural integrity against water dilution and pH change in an aqueous milieu. The notable stability of SFN-loaded particles is critical for maintaining structural integrity throughout the gastrointestinal tract during oral administration.

SFN has food effects and cancer patients are recommended to take SFN tablets without food. The components and pH of gastric fluid and intestinal fluid are different; thus, it is critical to test oral drugs in both gastric fluid and intestinal fluid. The dissolution of SFN granules was conducted by using a two-step dissolution method simulating physiological conditions. SFN granules remarkedly increased the dissolution rate compared to SFN powder in SIF (Figure 5). Coating SFN with the binary lipid mixture, the formation of SFN-loaded particles, the amorphous form, and the lipolysis of lipid components in the granules contributed to the dissolution enhancement.

Pharmacokinetic and tissue distribution studies confirmed that SFN granules enhanced oral absorption. SFN granules significantly increased blood concentration and tissue uptake compared to SFN powder (Figure 6 and Figure 7). There are several reasons for enhanced absorption. First is the increasing dissolution by SFN granules. Secondly, the lipid and surfactant in SFN granules could enhance the permeability of SFN. Thirdly, coating the stable binary lipid mixture on the SFN surface could increase the saturated solubility and local concentration of SFN. Finally, it is known that lipid formulations increase drug lymphatic uptake [24,25]. Indeed, the tissue distribution demonstrated that SFN granules increased lymphatic uptake by over 20-fold compared to SFN powder. SFN is a key treatment for renal cell carcinoma and unresectable hepatocellular carcinoma. According to the results of biodistribution (Figure 7), SFN granules greatly increased SFN concertation in liver over 6.6-fold and in kidney over 7.8-fold. Thus, SFN granules have potential to improve the efficacy of tumor inhibition because of the increased drug concentrations in tumor tissues.

In conclusion, new lipid-based SFN granules were prepared by coating a stable binary lipid mixture of Miglyol 812 and TPGS on the SFN surface. With 10% of DL, SFN granules demonstrated remarkable stability at room temperature over three months. SFN granules produced stable SFN-loaded particles upon contact with water. Compared to SFN powder, SFN granules increased dissolution rate and oral absorption. Moreover, the preparation of lipid-based SFN granules is simple and scalable. Thus, the new lipid-based SFN granules have the potential to enhance the systemic exposure of SFN.

## Figures and Tables

**Table 1:** Long-term stability of SFN granules and SFN-loaded particles (*n* = 3). Data are presented as mean ± SD. DL% and degradation% were measured for SFN granules. Entrapped SFN%, particle size and P.I. were measured for SFN-loaded particles that were produced once SFN granules mixed with water.

Parameters	Day 0	Two Weeks	One Month	Two Months	Three Months
Measured DL%	9.7 ± 0.1	9.5 ± 0.4	9.5 ± 0.3	9.2 ± 0.2	9.4 ± 0.2
Degradation%	0	0	0	0	0
Entrapped SFN%	99.9 ± 0.029	100 ± 0	100 ± 0	100 ± 0	99.9 ± 0.018
Particle size (nm)	145 ± 5	146 ± 1	147 ± 4	160 ± 8	162 ± 22
P.I.	0.266 ± 0.045	0.272 ± 0.028	0.29 ± 0.062	0.262 ± 0.05	0.268 ± 0.01

## Data Availability

Data is contained within the article.
